# Practical Notes on Diagnosis and Treatment

**Published:** 1908-03-07

**Authors:** 


Treatment.

### Practical Notes on Diagnosis and Treatment.

**Babinski's Sign.**

According to Dr. Rudolf, of Toronto, an extensor response, instead of the normal flexor movement of the toes, may be obtained in many healthy people if the test is applied while the individual is sleeping.

**Recurrent Pleural Effusion.**

To prevent the return of a pleural effusion, the injection of formalin has been adopted. After paracentesis, an ounce of glycerine containing 10 drops of formalin may be introduced into the pleural cavity.

**Chlorosis.**

In severe cases of this condition it is usually difficult or impossible to get a good result from iron unless the patient is at the same time ordered to take complete rest in bed.

**Double Cyanide Gauze.**

I have often regretted that the double cyanide of mercury and zinc is not more generally employed, especially in foreign countries. Bichloride of mercury must not be used with the gauze, because it forms with the double cyanide a triple compound which is both feebly germicidal and highly irritating.—*Lord Lister.*

**Interstitial Nephritis.**

In this disease there is often retention of chlorides without accompanying œdema. This can be shown by a daily estimation of the chlorides eliminated in the urine. A salt-free diet for a few days, or even weeks, is followed by continued elimination of chlorides, and often by great improvement in the patient's health.

**Lumbar Puncture.**

Lumbar puncture must only be practised on a patient who is in bed; and the patient must remain in bed for some hours after the operation. Otherwise vomiting, headache, convulsions, or vertigo may supervene. According to Babinski, lymphocytosis of the cerebro-spinal fluid is absolutely diagnostic of syphilis.

**Menière's Disease.**

Obstinate cases of this condition, even when all other remedies have failed to give relief, have been known to yield to the use of a seton. The seton may, perhaps, have to be worn for many months.

**Merycism.**

This name is applied to the habit of rumination when occurring in the human subject. A number of cases have been recorded, and in most of these the patients were in good general health.

**Mongolism.**

Children of this type nearly all practise tongue-sucking. The tongue appears large, and its mucous membrane has a sodden appearance and shows prominent papillæ and numerous fissures.

**High Blood Pressure.**

Experience shows that a strict milk diet or a lacto-farinaceous diet for a few weeks has an excellent effect on high arterial pressure and on the general health.—*Dr. Geo. Oliver.*

**Malignant Disease of the Viscera.**

Primary malignant disease of the lung or bladder is by no means always in its clinical aspect an urgent and distinctive process, and the symptoms resulting therefrom may fail altogether to suggest the serious conditions on which they, as a matter of fact, depend. Hence secondary or metastatic developments in such cases may readily fail to secure an accurate and ready interpretation.—*Dr. C. O. Hawthorne.*

**Diaphragmatic Pleurisy.**

The symptoms of this condition commence, as a rule, with severe abdominal pain, and often with a rigor; the temperature rises to 103° or 104°, and the pulse is rapid; the abdomen is rigid, and is tender to pressure, and the knees are drawn up. Hiccough is frequent, and nausea and vomiting are common. Altogether the symptoms are not unlike those of acute peritonitis.

